# Pancreatico-Gastrostomy: A Modified Two-Layered Technique

**DOI:** 10.7759/cureus.26227

**Published:** 2022-06-23

**Authors:** Ajay K Boralkar, Abdul Rafe, Anagha S Varudkar, Kunal Vikram Singh

**Affiliations:** 1 Surgical Oncology, Government Cancer Hospital, Aurangabad, IND

**Keywords:** pancreatic duct, pancreato-duodenal resections, popf, pancreatico-gastrostomy, periampullary carcinoma

## Abstract

Introduction: Pancreato-duodenal resections are commonly done for periampullary carcinomas. The outcome of the procedure is decided by pancreato-enterostomy. Pancreato-jejunostomy (PJ) has been employed largely as pancreato-enterostomy. Recently, there has been a renewed interest in pancreato-gastrostomy (PG). The debate continues on the choice of reconstruction.

Methods: A hundred cases of periampullary carcinoma were subjected to modified pancreatico-gastrostomy. The pancreatico-gastrectomy was evaluated by drain fluid amylase done on days 1, 3, and 5 post-operatively and clinical findings. The leaks were classified according to the International Study Group of Pancreatic Fistula (ISGPF) classification of biochemical leaks, postoperative pancreatic fistula (POPF) B and POPF C. The leaks were evaluated against pancreatic factors like duct diameter, consistency of the pancreas, the thickness of the pancreatic neck, and duct location.

Observations: Eighty percent of patients had no leaks. The biochemical leak was seen in 10% of cases. POPF B and C were observed at 5% each. Mortality was 3%. The diameter of the pancreatic duct of more than 3 mm and the firm consistency of the pancreas were favourable factors in the outcome of the anastomosis.

Conclusion: A modified pancreatico-gastrostomy technique appears to be technically feasible and safe. The leak rates and mortality appear to be low. We need a higher number of patients to confirm the efficacy of this modified pancreatico-enterostomy.

## Introduction

Pancreaticoduodenal resections have traditionally been used for periampullary tumours, including the head pancreas and duodenal tumours, which are mostly malignant. Pancreatico-enteric anastomosis remains the most significant factor in determining the procedure's outcome. Although the mortality rate of pancreatico-enterostomy has been reduced to 3-5% [1], morbidity from pancreatico-enterostomy continues to be variable, up to 30% [2]. The use of neither anastomotic stents [3] nor fibrin glue [4] for anastomosis has reduced the rates of postoperative pancreatic fistula (POPF). The debate over the two methods of pancreatico-enterostomy, namely pancreato-jejunostomy (PJ) and pancreatico-gastrostomy (PG), has been continuing for decades. However, PG has recently gained popularity in some areas [5]. The cumulative risk of fistula occurrence was reported to be lower in PG than in PJ [6]. Various techniques of PG have been reported in the literature, such as the pancreatic invagination technique, duct-to-mucosa anastomosis by using the entire stump of the pancreas, and duct-to-mucosa anastomosis with gastric partition. This study evaluated the PG procedure performed in our centre, a tertiary-care cancer hospital in suburban central India, with regard to the factors affecting the outcomes of anastomosis.

## Materials and methods

In total, 100 patients with periampullary carcinomas, including head pancreas, uncinate process, distal bile duct, and duodenum carcinomas, were operated on at the Government Medical College and Cancer Hospital, Aurangabad, India, from October 2014 to October 2020. The Institutional Ethics Committee approved the study protocol via letter-number Pharma/IEC-GMCA/565/2019. The patients were evaluated for signs and symptoms of local and metastatic diseases and serum biochemical parameters through the liver function tests, serum carbohydrate antigen 19-9, and carcinoembryonic antigen tests. A computed tomography scan was performed to assess the chest and abdomen. The bones and brains of symptomatic patients were evaluated. A positron emission tomography scan for staging was not performed for any patient. No preoperative biliary stents were placed. Operable patients were administered pylorus-preserving pancreaticoduodenectomy or classical Whipple’s procedure. Intraoperative findings were noted. Drains were placed around the PG (peri-PG drain) and hepaticojejunostomy sites. Postoperatively, patients were initiated with oral sips on day 1, and the diet was advanced as per patients’ tolerance. The peri-PG drain amount per day was noted. Drain fluid amylase values were estimated on postoperative days 1, 3, and 5. If the drained amount persisted after day 5 or the day 5 fluid amylase level was high, the day 7 fluid amylase level was estimated. Patients received subcutaneous somatostatin analogue of 100 μg three times a day for five to seven days. Antibiotics were initiated at anaesthesia induction and were continued for five days postoperatively or based on fever occurrence, culture results, or neutrophil counts. The peri-PG drain was removed one day after biochemical normalcy of the drain fluid or when it was <50 cc/day. Patients without complications were discharged on day 7. Histological findings were noted. The leaks were classified as POPF B or POPF C according to the modified International Study Group of Pancreatic Fistula (ISGPF) classification [7].

Operative procedure

A midline or extended Kocher’s incision was performed. Metastatic disease was ruled out. Hepatoduodenal lymphadenectomy was performed. The gallbladder was removed from the gallbladder fossa, and the common hepatic duct was divided at 5 mm above the cystic-hepatic duct junction. The common hepatic artery nodes and superior border of the pancreas lymph nodes were cleared. The portal vein was cleared of lymph nodes from the vena porta to the splenic vein. The duodenum was kocherised and posteriorly mobilised from the inferior vena cava, and a retroperitoneal margin was obtained. After devascularising, the proximal 15 cm of jejunum was divided; the proximal 5 cm of the superior mesenteric vein (SMV) was cleared of nodes, and the clearance continued up to the splenic vein-SMV junction. The pancreas was divided at the neck, and the distal remnant was mobilised for 3 cm. The head pancreas, along with the duodenum, was mobilised from the SMV and portal vein, clipping or coagulating small tributaries draining into the SMV and portal vein. The mesopancreas was divided along the lateral wall of the superior mesenteric artery, clipping or ligating the inferior pancreaticoduodenal artery at its origin.

PG procedure

**Figure 1:**
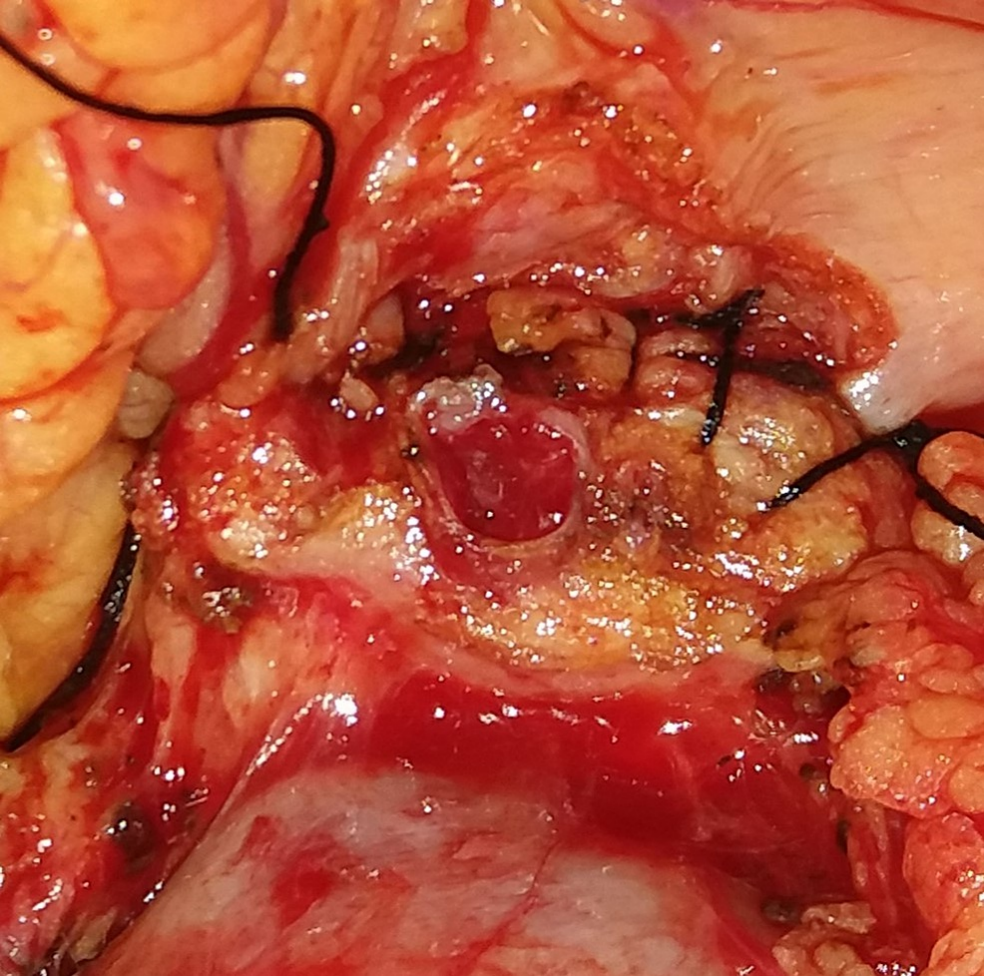
First layer: pancreatic parenchyma and the seromuscular layer of stomach

**Figure 2:**
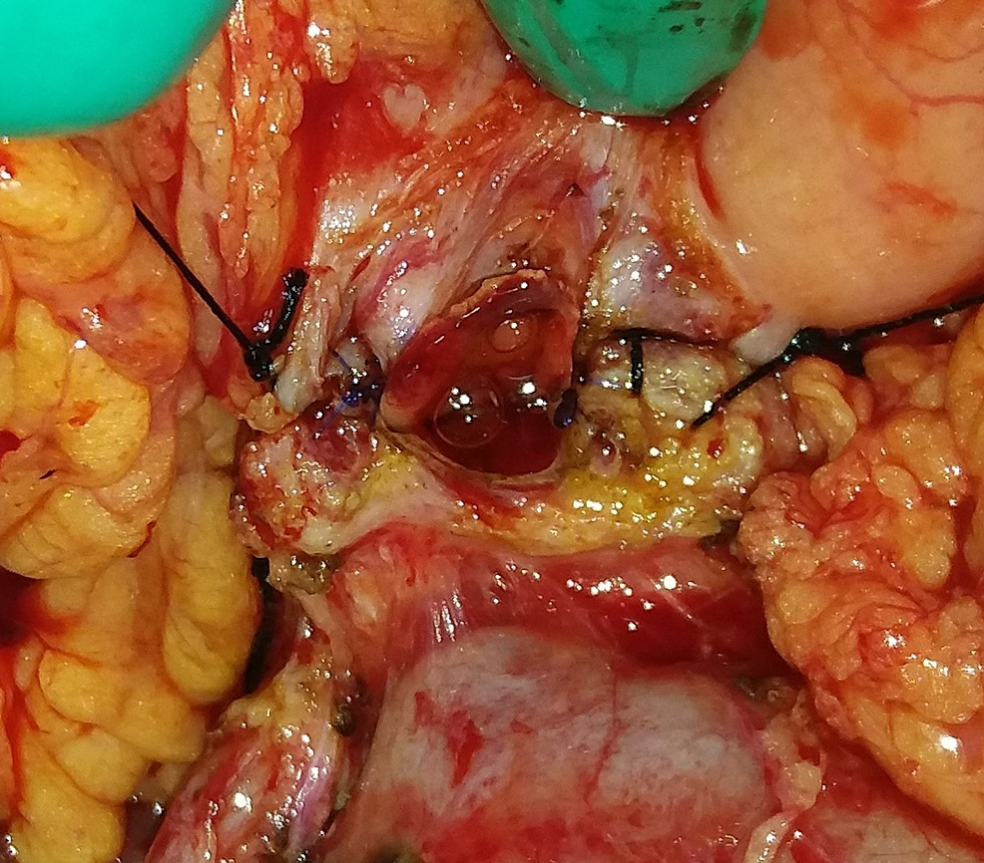
Second layer: pancreatic duct and gastric mucosa

**Figure 3:**
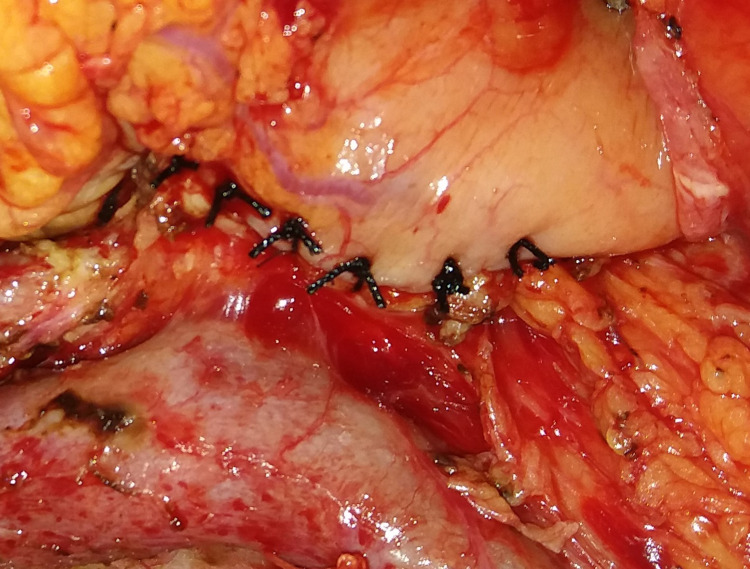
Completed pancreatico-gastrostomy

At a suitable part of the distal posterior wall of the stomach, a transverse incision equal to the size of the cut pancreatic remnant was made. The incision was deepened up to the mucosa. The upper and lower margins of the incision were raised as seromuscular flaps for a centimetre without breaching the mucosa. Button-holing of the mucosa was performed on the opposite side of the pancreatic duct. The parenchyma of the pancreas at the anterior cut margins was sutured to the upper seromuscular flap of the posterior gastric wall incision with interrupted ligatures of silk (2-0 or 3-0). The pancreatic duct mucosa was anastomosed to the hole in the gastric mucosa with simple interrupted sutures by using 5-0 polydiaxanone (PDS) or polypropylene knots outside. The posterior cut margin of the pancreatic parenchyma was then sutured to the distal flap of the gastric incision. No stents were placed through the anastomosis. A peri-PG suction drain was placed. Single-layer hepaticojejunostomy with PDS 4-0 and two-layer pylorojejunostomy with vicryl 2-0 were used to complete reconstruction. A subhepatic abdominal tube drain was placed. No feeding jejunostomy was placed. A normal saline wash was given, and the incision was closed in layers. Figures 1-3 show images of the first layer, the second layer, and the completed pancreatico-gastrostomy, respectively.

## Results

Biochemical leaks were noted but excluded from categorization as POPF. Eighty patients (80%) had no leak, 10 patients (10%) had a biochemical leak, 5 patients (5%) had POPF B and 5 patients (5%) had POPF C. Three patients (60%) of the five patients with POPF C succumbed even after re-exploration. The factors that were studied with respect to POPF were parenchymal consistency, pancreatic duct diameter, parenchymal thickness, and duct location.

Forty-three patients (43%) had a soft pancreas and 57 patients (57%) had a firm pancreas. Table 1 describes the distribution of pancreaticogastrostomy outcomes in relation to the consistency of the pancreas. The pancreas was adjudged as firm or soft in texture by the same surgeon.

**Table 1:** Pancreaticogastrostomy outcomes and consistency of pancreas POPF B: post-operative pancreatic fistula grade B; POPF C: post-operative pancreatic fistula grade C

Pancreaticogastrostomy outcomes	Consistency of the pancreas: soft	Consistency of the pancreas: firm
No leak	32	48
Biochemical leak	05	05
POPF B	02	03
POPF C	04	01
Total	43	57

The pancreatic duct diameter at the cut end of the pancreatic neck was evaluated at 3 mm or less and more than 3 mm (Table 2). One out of 56 (1.8%) patients had POPF when the duct diameter was more than 3 mm, while 9 out of 44 (20.5%) had POPF when the duct diameter was 3 mm or less. No POPF C was observed with ducts more than 3 mm in diameter.

**Table 2:** Pancreaticogastrostomy outcomes in relation to duct diameter POPF B: post-operative pancreatic fistula grade B; POPF C: post-operative pancreatic fistula grade C

Pancreaticogastrostomy outcomes	Duct diameter - 3 mm or less	Duct diameter - more than 3 mm
No leak	27	53
Biochemical leak	08	02
POPF B	04	01
POPF C	05	00
Total	44	56

As shown in Table 3, the thickness of the pancreas at the cut end of the neck was less than 1 cm in 55 patients (55%), and 1 cm or more in 45 patients (45%). Six patients (10.9%) out of 55 patients had POPF when the thickness was less than 1 cm and 4 out of 45 patients (8.9%) had POPF when the thickness was 1 cm or more.

**Table 3:** Pancreaticogastrostomy outcomes in relation to thickness at neck pancreas POPF B: post-operative pancreatic fistula grade B; POPF C: post-operative pancreatic fistula grade C

Pancreaticogastrostomy outcomes	Thickness at neck pancreas - less than 1 cm	Thickness at neck pancreas - 1 cm or more
No leak	44	36
Biochemical leak	05	05
POPF B	03	02
POPF C	03	02
Total	55	45

The centralized duct was not common in this study. The duct was located centrally in 21/100 (21%) and posteriorly in 79/100 (79%) cases. We did not have any cases of anteriorly decentralised ducts. POPF occurred in 4/21 (19%) with ducts that lay central, while 6/79 (7.6%) of those posteriorly placed had leaks (Table 4).

**Table 4:** Pancreaticogastrostomy outcomes in relation to location of duct POPF B: post-operative pancreatic fistula grade B; POPF C: post-operative pancreatic fistula grade C

Pancreaticogastrostomy outcomes	Duct location: central	Duct location: posterior
No leak	15	65
Biochemical leak	02	08
POPF B	02	03
POPF C	02	03
Total	21	79

## Discussion

Our study results have a 10% rate of POPF among the 100 patients operated on with pancreatico-gastrostomy. The comparison of rates of post-operative pancreatic fistula with pancreatico-jejunostomy and pancreatico-gastrostomy shows a favourable outcome with a two-layered anastomotic technique as compared with different series of pancreatico-jejunostomies. The study by Wada et al. [8] showed that 266 patients operated with pancreatico-jejunostomy had an 11.7% rate of POPF. Lyu et al. [9] found an 18.9% rate of POPF in a meta-analysis of 1099 patients who had pancreatico-jejunostomy surgery. In the study by Li et al. [10], 299 patients operated with pancreatico-jejunostomy had a 13.5% rate of POPF, while in the study by Polanco et al. [11], 150 patients operated with pancreatico-jejunostomy had a 17.3% rate of POPF. Similar to our study results, 10% POPF rates after pancreaticoduodenectomy have been described by Chen et al. [12].

The bone of contention for mortality and morbidity remains the post-operative pancreatic fistula and so the choice of pancreatico-enterostomy. It is reported by Cheng et al. [13] that for the choice of pancreatico-enterostomy, there is no statistically significant advantage of PG over PJ. The duct diameter, 3 mm or less, is significantly more associated with pancreatic fistula. According to Bing-yang et al. [14], fistulas occur in 10.3% of cases when the duct is larger than 3 mm and in 23.8% of cases when the duct is smaller. The larger duct lumen facilitates flow, avoiding stasis near the suture line. We noted that these ducts often have thicker walls than smaller ducts and hold sutures well. The seromuscular coat of the stomach provides a relatively tough envelope to the duct-gastric mucosa sutures. The neck of the pancreas is mobilized and anastomosis towards less curvature of the stomach keeps it tension free. We had only one fistula when the diameter was more than 3 mm.

Pancreatic texture plays an important role in the integrity of pancratico-gastrostomy. The firm pancreas owing to fibrosis makes reliable sutures with the seromuscular coat of the stomach. A systematic meta-analysis of pancreatoduodenectomies from seven institutions by Gio Xingjun et al. [15], found that the robustness of pancreato-enterostomy depends on fibrosis and firmness of the pancreas. The soft pancreas is more prone to tearing of anastomotic sutures, causing altered pancreatic exocrine function. In our series, the soft pancreas had a POPF rate of almost twice as compared to the firm pancreas. Also, the soft pancreas was indicative of elevated rates of POPF as concluded by Eshmuminov et al. [16]. However, using a modified technique of PG with two continuous hemistitch sutures placed in the mucosal and seromuscular layers of the posterior gastric wall, respectively, Feng Zhu et al. [17] reported POPF in only 2.2% of 96 cases with the soft pancreas.

The location of the pancreatic duct can be anterior, central, or posterior at the cut end of the neck pancreas. The main pancreatic duct de-centralization in the antero-posterior axis increases the leak rate, as was observed by Christina Ridolfi et al. [18]. The centralized duct offers duct to mucosa anastomosis secured by pancreatic parenchyma, while the de-centralized ducts have a part in their circumference where the anastomosis cannot be covered by the seromuscular part of the bowel and pancreatic parenchyma.

In our study, six patients (10.9%) out of 55 patients had POPF when the thickness was less than 1 cm, and four out of 45 patients (8.9%) had POPF when the thickness was 1 cm or more. Nikhil et al. [19] observed an increased rate of postoperative pancreatic fistula when the pancreatic thickness was less than 12 mm at the pancreatic neck.

## Conclusions

Pancreatico-gastrostomy is being used more than before as a pancreatico-enterostomy. The pancreatico-gastrostomy used is a novel, feasible, and safe procedure. The robust seromuscular coat of the stomach appears to offer more reliability and thus benefits in a two-layered anastomosis. The POPF rates are towards the lower end of the range quoted by most of the series. A firm pancreas and large duct diameter seem to be the most determining factors for the POPF in a pancreatico-gastrostomy. Though short-term results are encouraging, long-term effects like stricture, effects on the pancreatic remnant, and physiology of digestion need to be studied further. Larger sample studies with two-layer pancreatico-gastrostomy are needed to further confirm the reliability of this method.
